# Association of Genetic Variants with Postoperative Donor Artery Development in Moyamoya Disease: *RNF213* and Other Moyamoya Angiopathy-Related Gene Analysis

**DOI:** 10.1007/s12975-024-01248-7

**Published:** 2024-04-09

**Authors:** Seiei Torazawa, Satoru Miyawaki, Hideaki Imai, Hiroki Hongo, Hideaki Ono, Shotaro Ogawa, Yu Sakai, Satoshi Kiyofuji, Satoshi Koizumi, Daisuke Komura, Hiroto Katoh, Shumpei Ishikawa, Nobuhito Saito

**Affiliations:** 1 Department of Neurosurgery, Faculty of Medicine, The University of Tokyo, 7-3-1 Hongo, Bunkyo-Ku, Tokyo, 113-8655 Japan; 2 Department of Neurosurgery, Tokyo Shinjuku Medical Center, Tokyo, Japan; 3 Department of Neurosurgery, Fuji Brain Institute and Hospital, Shizuoka, Japan; 4 Department of Preventive Medicine, Graduate School of Medicine, The University of Tokyo, Tokyo, Japan

**Keywords:** Moyamoya disease, Bypass surgery, Collateral, Rare variant, *RNF213*

## Abstract

Robust postoperative bypass development is a characteristic of moyamoya disease (MMD); however, genetic factors mediating this phenomenon remain incompletely understood. Therefore, we aimed to elucidate the relationship between postoperative donor artery development and genetic variants. We retrospectively enrolled 63 patients (79 hemispheres) who underwent combined revascularization surgery. Postoperative development of the superficial temporal artery (STA), middle meningeal artery, and deep temporal artery (DTA) was assessed using the caliber-change ratio determined from magnetic resonance angiography measurements. We analyzed *RNF213* and 36 other moyamoya angiopathy-related genes by whole-exome sequencing and extracted rare or damaging variants. Thirty-five participants carried *RNF213* p.Arg4810Lys (all heterozygotes), whereas 5 had *RNF213* rare variants (RVs). p.Arg4810Lys was significantly associated with postoperative DTA development, while age at surgery, hypertension, and hyperlipidemia were inversely associated. Multiple regression analysis revealed that age and p.Arg4810Lys held statistical significance (*P* = 0.044, coefficient − 0.015, 95% confidence interval (CI) − 0.029 to 0.000 and *P* = 0.001, coefficient 0.670, 95% CI 0.269 to 1.072, respectively). Those with *RNF213* RV without p.Arg4810Lys exhibited a significant trend toward poor DTA development (*P* = 0.001). Hypertension demonstrated a significant positive association with STA development, which remained significant even after multiple regression analysis (*P* = 0.001, coefficient 0.303, 95% CI 0.123 to 0.482). Following Bonferroni correction for multiple comparisons, targeted analyses of *RNF213* and 36 moyamoya angiopathy-related genes showed a significant association of only *RNF213* p.Arg4810Lys with favorable DTA development (*P* = 0.001). A comprehensive analysis of *RNF213*, considering both p.Arg4810Lys and RVs, may provide a clearer prediction of postoperative DTA development.

**Supplementary Information:**

The online version contains supplementary material available at 10.1007/s12975-024-01248-7.

## Introduction

Moyamoya disease (MMD) is characterized by progressive stenosis at the terminal portion of the internal carotid artery and the development of collateral vessels at the base of the brain and other collateral networks, such as the leptomeningeal anastomosis and anastomoses between the branches of the external carotid artery and intracranial arteries [1, 2]. Another significant feature of MMD is the development of prominent synangiosis between the pial arteries and muscles, dura mater, and pericranium following surgical revascularization [1]. Owing to this characteristic, diverse variations of indirect revascularization surgery (e.g., encephalo-duro-arterio-synangiosis, and encephalo-duro-arterio-myo-synangiosis) have been considered effective for MMD and employed either as standalone procedures or in conjunction with the direct bypass (called “combined bypass”) [1, 3]. We mainly adopt a combined bypass approach in our practice, as per a recent study that demonstrated the efficacy of the combined bypass approach for facilitating the postoperative development of both direct and indirect collateral networks [4]. However, a detailed understanding of the factors contributing to the development of both direct bypass (blood flow from superficial temporal artery (STA)) and indirect bypass (blood flow from middle meningeal artery (MMA) and deep temporal artery (DTA)) could lead to the selection of more optimal surgical strategies.

Age is widely recognized as a contributory factor in postoperative bypass development in patients with MMD, as neovascularization routinely develops more robustly in younger patients, whereas adults often show suboptimal development of neovascularization [5–7]. Despite the reported associations with other preoperative factors, such as hyperlipidemia, disease stage, and hemorrhagic onset, there is no established consensus on these relationships [5, 8, 9].

Regarding genetic factors, *RNF213* c.14429G > A (p.Arg4810Lys) is a significant risk variant for MMD [10, 11]. Controversy exists as several recent studies have reported a significant association between p.Arg4810Lys and postoperative bypass development [5, 8, 12], whereas another report has reported no such association [13]. Reports on the association between postoperative bypass development and genetic variants are limited, and to date, only associations with p.Arg4810Lys have been analyzed. A recent report indicated the possibility that *RNF213* rare variants (RV) other than p.Arg4810Lys may act as modifying factors in the clinical phenotype [14]. Additionally, several other genes, known as moyamoya angiopathy-related genes, constitute potential genetic factors that contribute to the vascular phenotype characteristics of MMD or moyamoya syndrome [15, 16]. Considering the literature, these *RNF213* RVs and the other genes could also be associated with postoperative bypass development. Therefore, we conducted targeted gene analysis of *RNF213* and other moyamoya angiopathy-related genes to elucidate the genetic factors associated with bypass development after combined bypass surgery.

## Methods

### Participants

In this retrospective cohort study, we recruited patients with MMD who agreed to undergo genetic analysis and combined bypass surgery, using both direct and indirect bypass methods, at our institution between October 2011 and December 2022. This study was approved by the research board of our institution (approval number: G10026; approval date: September 12, 2011). Written informed consent was obtained from all participants or the legal guardians of patients aged < 18 years. MMD was diagnosed based on the latest criteria of the Research Committee on Moyamoya Disease [17]. For the surgical procedure, a combined bypass of the direct STA–middle cerebral artery (MCA) anastomosis with encephalo-myo-synangiosis (EMS) was performed as previously reported [18], with some minor modifications. Cases exhibiting substantial contiguous brain damage in the frontotemporal craniotomy region before surgery were excluded from bypass procedures. The reporting of this study followed STROBE guidelines (Online Resources).

### Clinical and Imaging Data Collection

The clinical data shown in Table 1 were collected (for the diagnostic criteria of the medical histories, see Online Resources). Hemorrhagic symptoms included intracerebral hemorrhage, intraventricular hemorrhage, and subarachnoid hemorrhage. For cerebrovascular characteristics, we assessed time-of-flight magnetic resonance angiography (MRA) images, including Suzuki grade and PCA involvement, which was defined as a > 50% occlusion or stenosis in segments P1–P3. In cases where preoperative single-photon emission computed tomography (SPECT) imaging was used to assess the quantitative values of regional cerebral blood flow (rCBF), the ratios of the rCBF in the MCA territory to the ipsilateral cerebellum were evaluated.

**Table 1:** Basic characteristics of all 79 hemispheres

	All hemispheres (*n* = 79)	*RNF213* GG (*n* = 33, 41.8%)	*RNF213* GA (*n* = 46, 58.2%)	*P*-value
Sex (female)	52 (65.8%)	21 (63.6%)	31 (67.4%)	0.729
Age at surgery, median (IQR)	41 (31.5–50)	40 (32–49)	42.5 (27–51.8)	0.937
Hypertension	26 (32.9%)	11 (33.3%)	15 (32.6%)	0.946
Diabetes mellitus	4 (5.1%)	1 (3.0%)	3 (6.5%)	0.636
Dyslipidemia	15 (19.0%)	10 (30.3%)	5 (10.9%)	**0.030**
Smoking	25 (31.6%)	14 (42.4%)	11 (23.9%)	0.081
Family history of MMD	11 (13.9%)	2 (6.1%)	9 (19.6%)	0.109
PCA involvement	15 (19.0%)	4 (12.1%)	11 (23.9%)	0.188
Suzuki grade	3 (3–3)	3 (3–3)	3 (3–3)	0.798
Preoperative symptoms				
TIA	44 (55.7%)	17 (51.5%)	27 (58.7%)	0.526
Infarction	24 (30.4%)	10 (30.3%)	14 (30.4%)	0.990
Hemorrhage	11 (13.9%)	6 (18.2%)	5 (10.9%)	0.512
*RNF213* rare variants	5 (6.3%)	5 (15.2%)	0 (0.0%)	**0.011**

The bold values indicate *P* < 0.05

*GA* heterozygote of p.Arg4810Lys, *GG* wild type of p.Arg4810Lys, *IQR* interquartile range, *MMD* moyamoya disease, *PCA* posterior cerebral artery, *TIA* transient ischemic attack

### Evaluation of the Postoperative Development of Direct and Indirect Collaterals

Postoperative donor artery caliber changes were assessed with MRA source images, which has been reported to correlate with postoperative bypass flow development observed on digital subtraction angiography (DSA) [19]. The development of STA reflects the development of direct bypass, while the development of MMA and DTA reflects the development of indirect bypass. The calibers of STA, MMA, and DTA were measured both pre- and postoperatively, using the method reported by Uchino et al. (Fig. S1) [19]. Then, caliber-change ratios (CCR) for each artery were calculated by dividing the postoperative caliber by the preoperative caliber. For the postoperative evaluations, we primarily used imaging performed between 6 months and 1 year after surgery. However, in cases where images from this period were unavailable for various reasons, the subsequently available images were used. Measurements of vessel calibers were assessed by two experienced neurosurgeons (T.S. and O.S.), who were blinded to the genotyping results and clinical information, and the mean values of the vessel calibers measured separately were adopted.

### Whole-Exome Sequencing (WES) and Targeted Gene Analysis

WES was conducted for all participants, and Twist Comprehensive Exome Panel Kit (Twist Bioscience, South San Francisco, CA, USA) was used. Sequencing data were generated using NovaSeq6000 (Illumina, San Diego, CA, USA) and a 150 bp paired-end sequencing protocol across rapid-flow cell lanes. We confirmed that the quality was not judged as “fail” in the FastQC. Alignment to the human reference genome (Genome Reference Consortium Human Build 38 (GRCh38) [hg38]) and variant detection were performed using Clara Parabricks 3.8.0 implementation of the Burrows–Wheeler Aligner and HaplotypeCaller, respectively. Variants that passed and were annotated as “PASS” in the VCF file were analyzed. We extracted variants present in the target genes, including *RNF213* and 36 other genes (previously reported as syndromic or primary moyamoya angiopathy-related genes; Table S1).

For each extracted variant, the allele frequency was analyzed using the Genome Aggregation Database (gnomAD) based on the entries in dbSNP (https://www.ncbi.nlm.nih.gov/snp/), and variant annotation was performed using ANNOVAR (https://annovar.openbioinformatics.org/en/latest/, updated on Mar 15, 2023). The variant was defined as “rare” when its minor allele frequency was < 0.01 in gnomAD. It was defined as “damaging” only if it met both of the following criteria simultaneously: (1) its combined annotation-dependent depletion score (GRCh38-v1.6) was > 20 and (2) it was categorized as “deleterious/damaging or probably deleterious/damaging” by two or more of the following four prediction tools: Sorting intolerance from Tolerant (SIFT), PolyPhen-2, MutationTaster, and Protein Variation Effect Analyzer (PROVEAN). We extracted variants that met the above criteria for being either “rare variants (RV)” or “damaging variants (DV).”

### Statistical Analysis

All statistical analyses were performed using SPSS version 26 (IBM, Armonk, NY, USA). The Mann–Whitney *U* test was used to compare the medians between two independent groups for continuous data. The chi-square or Fisher’s exact test was used to compare the proportions of categorical variables. Linear regression analysis was applied when both the independent and dependent variables were continuous. Multiple regression analysis was used for the multivariate analysis of continuous outcomes. Statistical significance was defined at *P* < 0.05.

## Results

Among the 63 participants (79 operated hemispheres), 41 (65.1%) were female, 35 (55.6%) were p.Arg4810Lys heterozygotes (GA), 28 (44.4%) were wild type (GG), and none were homozygotes. As regards *RNF213* RV, five RVs were detected in five patients (7.9%), all of whom were GG of p.Arg4810Lys; no non-rare DVs were identified (Table S2). The RVs were p.Gly2440Asp, p.Asp2554Glu, p.Arg2704Gln, p.Met3666Thr, and p.Glu4950Asp (two of which fulfilled the current criteria for “damaging”: p.Arg2704Gln and p.Met3666Thr; Table S3). We analyzed 79 hemispheres that underwent combined bypass surgery, and their basic characteristics are summarized in Table 1. The median age of the patients at the time of surgery was 41 years. Preoperative hemispheric symptoms included transient ischemic attacks (TIA) in 44 patients (55.7%), cerebral infarctions in 24 (30.4%), and hemorrhages in 11 (13.9%). The mean duration from surgery to postoperative donor artery evaluation was 270 days. Regarding long-term postoperative clinical outcomes, there were no instances of patients experiencing an ipsilateral recurrent stroke, except for one case in which a patient suffered a symptomatic infarction in the ipsilateral frontal lobe one year after surgery.

### Analysis of Factors Associated with Postoperative Development of Collaterals

We calculated the CCR of each donor artery and analyzed the significant factors associated with the CCR (Table 2). We investigated individual underlying factors, such as sex, age at surgery, medical history, smoking history, family history of MMD, and p.Arg4810Lys. Hemispheric factors included PCA involvement, Suzuki grade, hemispheric preoperative symptoms, and the reciprocal relationship between the postoperative development of direct and indirect bypass.

**Table 2:** Analysis of factors associated with the caliber-change ratio (CCR) of STA, MMA, and DTA

	CCR of STA		CCR of MMA		CCR of DTA	
	Median (IQR)	*P-*value	Median (IQR)	*P*-value	Median (IQR)	*P*-value
Sex		0.102		0.852		0.649
Male	1.06 (0.86–1.28)		1.26 (1.05–1.38)		2.26 (1.60–3.36)	
Female	1.12 (1.02–1.43)		1.20 (1.02–1.44)		2.24 (1.44–2.84)	
Age at surgery*		0.823 (*B* 0.001)		0.164 (*B* − 0.004)		**0.001** (*B* − 0.022)
Hypertension		**0.007**		0.050		**0.007**
Yes	1.33 (1.06–1.56)		1.11 (0.93–1.34)		1.65 (1.39–2.26)	
No	1.06 (0.88–1.26)		1.26 (1.08–1.45)		2.44 (1.94–3.27)	
Diabetes mellitus		0.442		0.112		0.871
Yes	0.99 (0.90–1.15)		1.43 (1.33–1.52)		2.01 (1.85–2.35)	
No	1.10 (0.95–1.40)		1.22 (1.02–1.41)		2.26 (1.51–3.90)	
Dyslipidemia		0.147		0.435		**0.027**
Yes	1.45 (1.03–1.62)		1.22 (0.84–1.38)		1.57 (1.30–2.44)	
No	1.09 (0.91–1.34)		1.23 (1.04–1.44)		2.31 (1.75–3.25)	
Smoking		0.870		0.354		0.487
Yes	1.06 (0.91–1.41)		1.34 (1.05–1.42)		2.32 (1.39–2.73)	
No	1.10 (0.94–1.39)		1.19 (1.02–1.42)		2.27 (1.60–3.19)	
Family history of MMD		0.388		0.871		0.153
Yes	1.81 (1.00–1.44)		1.23 (1.11–1.38)		3.20 (1.53–3.67)	
No	1.06 (0.92–1.40)		1.22 (1.02–1.44)		2.22 (1.54–2.82)	
PCA involvement		0.409		0.736		0.901
Yes	1.16 (0.95–1.44)		1.27 (1.15–1.37)		2.18 (1.99–2.69)	
No	1.08 (0.91–1.37)		1.20 (1.02–1.44)		2.25 (1.40–3.18)	
Suzuki grade≧4		0.346		0.229		0.882
Yes	1.16 (1.05–1.42)		1.35 (1.23–1.54)		2.18 (1.66–3.00)	
No	1.08 (0.91–1.39)		1.20 (1.02–1.39)		2.25 (1.54–3.01)	
Preoperative symptoms						
TIA		0.193		0.493		0.820
Yes	1.15 (1.02–1.41)		1.20 (1.02–1.37)		2.26 (1.73–3.08)	
No	1.06 (0.88–1.32)		1.23 (1.06–1.45)		2.24 (1.39–3.06)	
Ischemia		0.962		0.433		0.324
Yes	1.06 (0.90–1.45)		1.27 (1.08–1.45)		2.12 (1.33–2.72)	
No	1.11 (0.95–1.38)		1.22 (10.2–1.38)		2.31 (1.70–3.21)	
Hemorrhage		0.053		0.955		0.325
Yes	0.98 (0.83–1.11)		1.24 (0.99–1.40)		2.73 (1.59–3.33)	
No	1.12 (1.00–1.41)		1.22 (1.02–1.42)		2.22 (1.53–2.95)	
Direct bypass development* (CCR of STA)		NA		0.431 (*B* − 0.093)		0.277 (*B* 0.047)
Indirect bypass development* (CCR of MMA or DTA†)		0.144 (*B* − 0.064)		NA		NA
*RNF213* p.Arg4810Lys		0.299		0.952		**0.001**
Yes (GA)	1.14 (0.92–1.41)		1.22 (1.03–1.41)		2.44 (2.01–3.34)	
No (GG)	1.06 (0.98–1.22)		1.22 (0.98–1.44)		1.74 (1.33–2.66)	

Bold values indicate *P* < 0.05

“B” represents the unstandardized regression coefficient

^*^Regression analysis

^†^For analysis, the larger value of CCR between MMA and DTA was used

*GA* heterozygote of p.Arg4810Lys, *GG* wild type of p.Arg4810Lys, *IQR* interquartile range, *MMD* moyamoya disease, *NA* not applicable, *TIA* transient ischemic attack

**Fig. 1:**
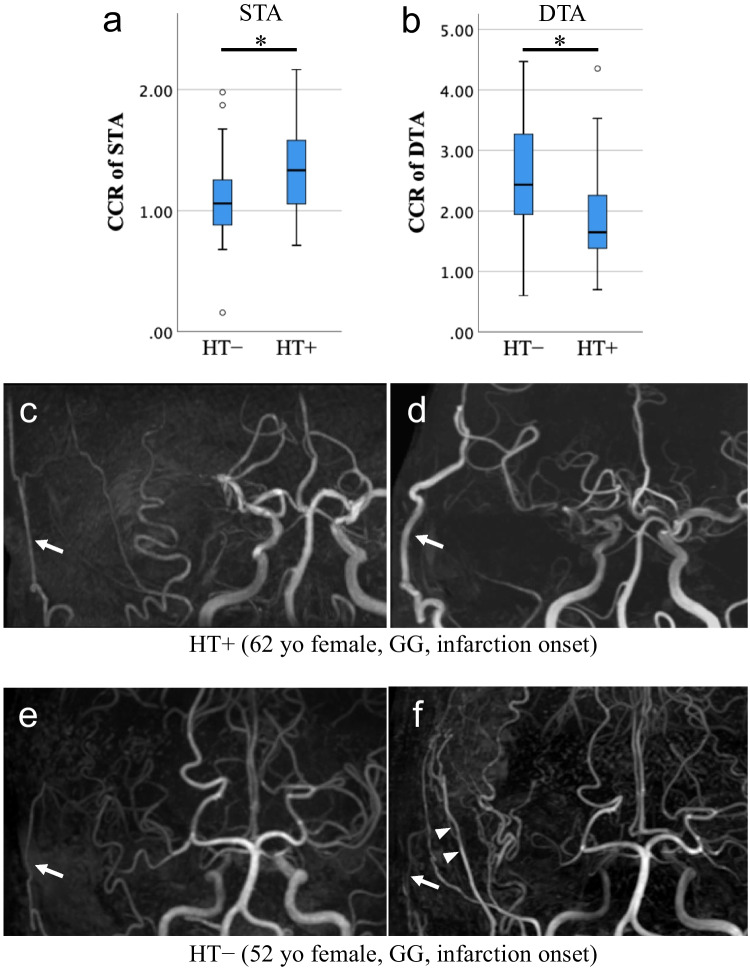
Effect of hypertension on the caliber-change ratio (CCR) of DTA and STA. The association between the CCR of STA and DTA and hypertension is shown in the boxplots (**a, b**). The CCR of STA is higher in patients with hypertension, whereas CCR of DTA is lower in those with hypertension; both with a *P*-value of 0.007. Images (**c–f**) show the preoperative and postoperative MRA of representative cases: one with hypertension (**c, d**) and another without hypertension (**e, f**). These two patients were of comparable age, female, without *RNF213* p.Arg4810Lys, and both presented with preoperative infarctions. In image **d,** the STA (indicated by an arrow) is well-developed, whereas the DTA is scarcely developed. In image **f,** the STA (indicated by an arrow) is patent but underdeveloped, whereas the DTA (indicated by an arrowhead) shows good development. The *P*-value was calculated using the Mann–Whitney *U* test. *P < 0.01. GG, wild type of p.Arg4810Lys; HT, hypertension

**Fig. 2:**
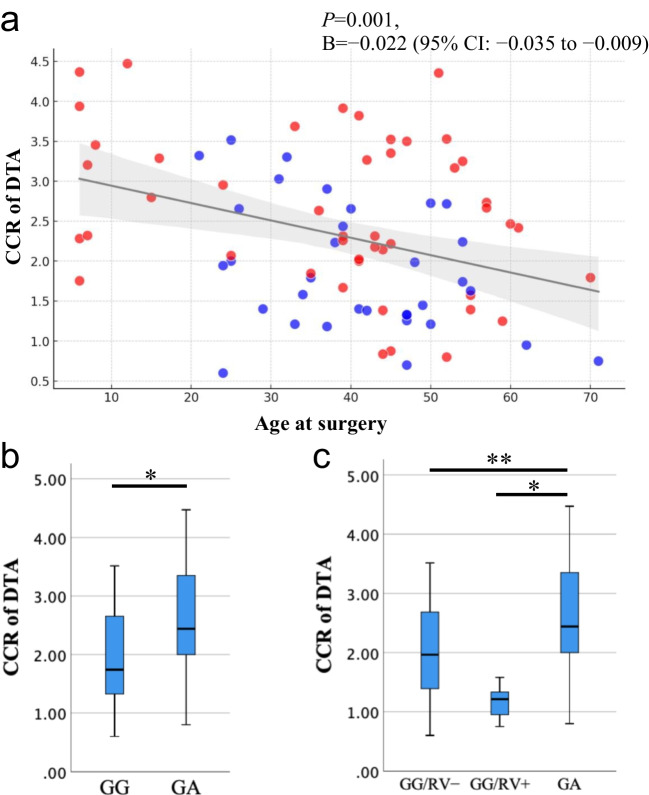
Statistically significant risk factors for the caliber-change ratio (CCR) of DTA, and comparison among genotypes of *RNF213*. **a **A scatter plot and regression analysis result showing the relationship between age at surgery and the CCR of DTA. Red dots represent the cases with GA, while blue dots indicate those with GG. *P*-values and regression coefficients were determined by regression analysis. Pearson’s correlation coefficient was − 0.353. “B” represents the unstandardized regression coefficient, which was − 0.022 (95% confidence interval − 0.035 to − 0.009). **b** A boxplot illustrating the association between the CCR of DTA and *RNF213* p.Arg4810Lys. *P*-value were computed using the Mann–Whitney *U* test. *P < 0.005. **c** A boxplot comparing the CCR of DTA among three genotype groups: GG/RV − , GG/RV + , and GA. *P*-values were determined using the Kruskal–Wallis test. *P < 0.005, **P < 0.05 followed by Bonferroni correction. GA, heterozygote of p.Arg4810Lys; GG, wild type of p.Arg4810Lys; RV, rare variant

Hypertension was significantly associated with the CCR of STA (*P* = 0.007). Regarding the CCR of DTA, p.Arg4810Lys showed a positive association (*P* = 0.001), whereas age at surgery, hypertension, and hyperlipidemia were negatively associated (*P* = 0.001, 0.007, and 0.027, respectively; associations of hypertension with the CCR of STA and DTA are shown in Fig. 1). No factor was associated with the CCR of MMA. Regarding the relationship between the CCR of STA and hypertension, even after adjustment (multiple regression analysis) for age at surgery, hypertension remained statistically significant (*P* = 0.001, coefficient 0.303, 95% confidence interval (CI) 0.123 to 0.482; Table 3). Following the univariate analyses for the CCR of DTA, a subsequent multiple regression analysis revealed that only age at surgery and p.Arg4810Lys retained statistical significance (*P* = 0.044, coefficient − 0.015, 95% CI − 0.029 to 0.000 and *P* = 0.001, coefficient 0.670, 95% CI 0.269 to 1.072, respectively; Table 3 and Fig. 2a, 2b). Meanwhile, there was no significant association between the preoperative Suzuki grade and preoperative symptoms with the CCR of any vessel (Table 2). Furthermore, although the analysis was limited to cases for which SPECT data were available, the preoperative rCBF reduction in the MCA territory was not significantly associated with the genotype of *RNF213* or with the bypass development (Tables S4 and S5). These findings suggest that the outcomes of our study were not significantly influenced by preoperative hemodynamic compromise.

**Table 3:** Multiple regression analysis of factors associated with the caliber-change ratio of STA and DTA

	*P-*value	Coefficient *B* (95% CI)
Caliber-change ratio of STA		
Age at surgery	0.177	− 0.004 (− 0.009 to 0.002)
Hypertension	**0.001**	0.303 (0.123 to 0.482)
Caliber-change ratio of DTA		
Age at surgery	**0.044**	− 0.015 (− 0.029 to 0.000)
Hypertension	0.132	− 0.370 (− 0.854 to 0.114)
Dyslipidemia	0.979	0.007 (− 0.559 to 0.574)
*RNF213* p.Arg4810Lys	**0.001**	0.670 (0.269 to 1.072)

The bold values indicate *P* < 0.05

*CI* confidence interval

### Association of RV and DV with Postoperative Collaterals: Targeted Analysis on RNF213 and Other Moyamoya Angiopathy-Related Genes

RVs and DVs were extracted from the WES results based on the earlier definitions and focusing on *RNF213* as well as the other 36 moyamoya angiopathy-related genes.

For *RNF213*, five RVs were identified in patients with GG, as previously described. A boxplot charting the CCR of DTA across the GG/RV − , GG/RV + , and GA groups revealed significant differences between the GA and the GG/RV + group (*P* = 0.001, corrected significance level followed by Bonferroni correction: *P* < 0.017; Fig. 2c).

**Fig. 3:**
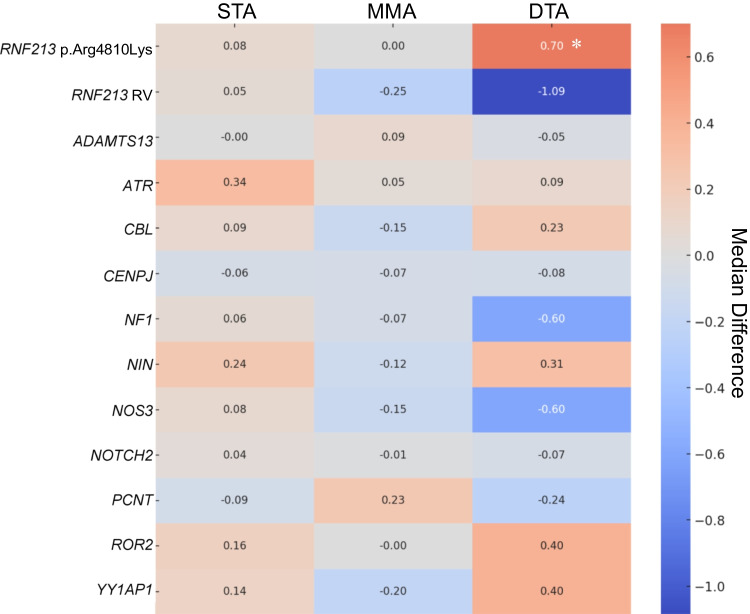
Heatmap representing the association of genetic variants with the caliber-change ratio of STA, MMA, and DTA. Values represent the differences in median CCR of each vessel between the groups with and without variants. * indicates statistical significance, even after Bonferroni correction (corrected significance level: *P* < 0.0038)

The moyamoya angiopathy-related genes *ACTA2*, *PTPN1*, *SOS1*, *HRAS*, *SMARCAL1*, *CECR1*, *SAMHD1*, *HBB*, and *EVL* did not have RVs or DVs. Among the remaining 27 moyamoya angiopathy-related genes, we identified 11 genes that exhibited either RV or DV in more than five hemispheres (for information on the variants detected in these 11 genes, see Table S6). The Mann–Whitney U test was used to determine whether the presence of *RNF213* p.Arg4810Lys, *RNF213* RVs, or these 11 genes was associated with the CCR of each vessel. Intergroup differences in the median CCR in groups with and without variants were visualized using a heatmap (Fig. 3). Following Bonferroni correction for multiple comparisons in each vessel, no statistically significant genes remained in STA or MMA. However, as for DTA, *RNF213* p.Arg4810Lys was significantly associated with an increased CCR (*P* = 0.001, corrected significance level followed by Bonferroni correction: *P* < 0.0038). *RNF213* RVs demonstrated a more pronounced negative association with the CCR of DTA than with any other gene, although they did not reach significance after correction (*P* = 0.004).

## Discussion

In this study, we performed WES and targeted analyses for *RNF213* as well as previously reported moyamoya angiopathy-related genes. The current findings indicate a pronounced association between *RNF213* and the postoperative development of DTA after combined bypass surgery, suggesting that p.Arg4810Lys can enhance DTA development and that *RNF213* RVs may reduce it. Furthermore, we found that younger age at surgery was associated with better DTA development. We also found that hypertension could be associated with altered vascular development, characterized by reduced DTA development and enhanced STA development.

Based on the current results, optimal surgical approaches can be determined before surgery. First, younger patients can be expected to have better DTA development after EMS. Even in older patients, p.Arg4810Lys still facilitates DTA development, indicating that combining EMS with direct bypass could be a more effective surgical approach in patients with this variant. Secondly, although DTA development tends to be poor in patients with hypertension, STA development may be significantly better. Thus, an emphasis on direct bypass over indirect bypass can be warranted in patients with hypertension.

p.Arg4810Lys is associated with certain MMD phenotypes. Carriers of this variant are more likely to exhibit PCA involvement [20, 21] and be more susceptible to cerebral ischemic symptoms [21–23]. Additionally, several reports have suggested the association of p.Arg4810Lys with the development of non-operative or postoperative collaterals. Ge et al. reported significant postoperative bypass development in p.Arg4810Lys carriers across cohorts including direct, indirect, and combined bypass surgeries [5]. Similarly, Ito et al. and Kawabori et al. reported enhanced development of indirect bypass from DTA in both adult and pediatric MMD patients with p.Arg4810Lys [8, 12]. In contrast to these reports, Zheng et al. asserted that, in a cohort of pediatric patients with MMD who underwent indirect bypass, there was no significant association between p.Arg4810Lys and postoperative bypass development [13]. In the context of non-operative collaterals, Kim et al. reported, albeit in a small cohort, that compared to those without the variant, adult MMD patients with p.Arg4810Lys exhibited poorer development of leptomeningeal collaterals [24]. Moreover, Ge et al. noted that while p.Arg4810Lys carriers exhibit poor development of the leptomeningeal collateral network, they demonstrate robust collateral development from branches of the external carotid artery [25]. By integrating these previous findings and the result of this study, it can be inferred that, due to poor leptomeningeal collateral development, p.Arg4810Lys carriers might develop enhanced bypass flow to compensate for ischemic conditions. In support of this hypothesis, animal studies have shown enhanced angiogenesis following hindlimb ischemia in *RNF213* knockout mice [26]. Although *RNF213* knockout mouse models have not shown significant cerebrovascular anomalies like MMD, and the pathophysiological mechanisms by which *RNF213* affects MMD remain unclear [16], further research is needed in the future to elucidate this intriguing relation between p.Arg4810Lys and enhanced angiogenesis.

In addition to p.Arg4810Lys, associations of *RNF213* RVs with certain MMD phenotypes have been reported [27–29]. Hara et al. reported that in pediatric-onset MMD undergoing indirect bypass surgery, patients without p.Arg4810Lys exhibited poorer postoperative outcomes compared to those with p.Arg4810Lys. Moreover, these p.Arg4810Lys-negative patients frequently had *RNF213* RVs (eight of 25 patients) [28]. Our finding of a potential relationship between *RNF213* RVs and poor postoperative DTA development may provide an explanation for their results. Notably, most of the *RNF213* RVs (four of five RVs) we identified in our cohort demonstrated higher CADD scores than p.Arg4810Lys, suggesting more severe functional effects on the RNF213 protein. This study is the first to suggest that *RNF213* variants, other than p.Arg4810Lys, may be associated with bypass development. Despite the preliminary nature of our findings, they raise the possibility that sequencing all variants of *RNF213* may offer predictive value for postoperative indirect bypass development.

Furthermore, we analyzed potential genes other than *RNF213* that were related to moyamoya angiopathy. To the best of our knowledge, this is the first study to investigate the association between postoperative bypass development and the variants of genes other than *RNF213*. Besides *RNF213*, no other gene was significantly associated with postoperative donor artery development. The moyamoya angiopathy-related genes we analyzed have been implicated in processes such as vascular remodeling, actin remodeling, the cell cycle, and the mitogen-activated protein kinase (MAPK) pathway [15, 16]. Thus, they have potential roles in vascular phenotypes. For instance, abnormal angiogenesis has been observed in *BRCC3*-knockdown zebrafish [30], and recent findings suggest that RVs in *ANO1* may impact cell membrane potential, thereby potentially influencing the function of vascular smooth muscle and endothelial cells [31]. Although analyses focused on *RNF213* p.Arg4810Lys have been the mainstream approach in MMD, it seems increasingly important to accumulate comprehensive phenotype–genotype analyses, considering the potential implications of these moyamoya angiopathy-related genes.

Regarding the association with patient characteristics other than genetic factors, two significant findings were evident from the multivariate analysis in this study: younger age and hypertension were associated with better DTA and STA development, respectively. The associations of Suzuki grade or hemorrhagic onset with postoperative collateral development, as previously reported [4, 5], were not confirmed in our study. The association between age and the development of indirect bypass was consistent with previous reports [5–7]. Few reports have analyzed the relationship between hypertension and postoperative bypass development. Among them, Ito et al. uniquely analyzed postoperative direct or indirect bypass separately in a cohort with combined bypass; however, no significant association with hypertension was demonstrated [8]. Although historical analyses have often underrepresented the association between the development of STA after combined bypass and hypertension, it is important to emphasize the need for further studies. By establishing a clearer relationship between postoperative bypass development and factors such as age or hypertension, we can make considerable contributions to determining more optimal surgical strategies.

This study had several limitations. First, this was a retrospective cohort study, which inherently introduced a selection bias, and the sample size was relatively small. We focused only on patients in whom WES could be performed and did not register cases continuously. This study design likely contributed to the comparatively low proportion of *RNF213* p.Arg4810Lys heterozygotes observed in our study, as compared to the higher frequencies reported in the existing literature. Therefore, it is crucial to interpret our findings with an understanding of this limitation, as it may influence the generalizability and applicability of our results. Second, we did not analyze genome-wide genes other than *RNF213*. Our analysis was limited to genes that were previously reported to be associated with moyamoya angiopathy. Third, our evaluation was based solely on the changes in the diameter of donor arteries. We did not analyze blood-flow distribution on DSA or perform hemodynamic evaluation on SPECT because these procedures were not performed in all cases. Additionally, it is important to note that our analysis did not include detailed postoperative outcomes. These outcomes include asymptomatic minor strokes that may have been incidentally detected on follow-up MRI, as well as subjective symptoms that are not apparent in follow-up imaging. The only exception to this is the single case that suffered from a symptomatic recurrent infarction, which is described in the Results section. Lastly, the exact pathophysiological mechanism by which *RNF213* variants influence donor artery development remains unknown, necessitating further functional analyses to elucidate this pathology in future studies.

## Conclusion

In targeted gene analysis, including previously reported moyamoya angiopathy-related genes, *RNF213* was identified as the most influential gene related to postoperative DTA development. p.Arg4810Lys is significantly associated with enhanced DTA development, whereas *RNF213* RVs may exert inhibitory effects on DTA development. The profiling of *RNF213* p.Arg4810Lys and RVs can enable the stratification and prediction of the outcomes of indirect revascularization procedures; thus, a comprehensive genetic analysis of *RNF213* is crucial.

## Supplementary Information

Below is the link to the electronic supplementary material.

Supplementary file1 (DOC 84 KB)

Supplementary file2 (DOCX 1546 KB)

## Data Availability

The data supporting the findings of this study are available from the corresponding author upon reasonable request from any investigator.

## Abbreviations

DTA: Deep temporal artery
GA: Heterozygote of p.Arg4810Lys
GG: Wild type of p.Arg4810Lys
IQR: Interquartile range
MMA: Middle meningeal artery
MMD: Moyamoya disease
NA: Not applicable
rCBF: Regional cerebral blood flow
SPECT: Single-photon emission computed tomography
STA: Superficial temporal artery
TIA: Transient ischemic attack
WES: Whole-exome sequencing
