# Supplementary material for: Association of Genetic Variants with Postoperative Donor Artery Development in Moyamoya Disease: RNF213 and Other Moyamoya Angiopathy-Related Gene Analysis
Source: Transl Stroke Res. 2024 Apr 9;16(3):679–89. doi: 10.1007/s12975-024-01248-7 (PMC12045808; doi:10.1007/s12975-024-01248-7)
Supplement: Supplementary file 2 — Supplementary file2 (DOCX 1546 KB) [file 12975_2024_1248_MOESM2_ESM.docx]

**Supplemental Material**

**Supplemental Methods**

**Diagnostic criteria for each underlying disease**

The diagnostic criteria for each underlying disease were as follows:

- Hypertension: Systolic blood pressure ≥140 mmHg or use of antihypertensive drugs.
- Diabetes mellitus: Fasting blood glucose >126 mg/dL, occasional blood glucose >200 mg/dL, or use of hypoglycemic drugs.
- Dyslipidemia: Fasting low-density lipoprotein cholesterol ≥140 mg/dL, high-density lipoprotein cholesterol <40 mg/dL, triglyceride >150 mg/dL, or oral treatment with lipid-lowering drugs.

**Figure S1** Postoperative time-of-flight MRA and its source images

**a:** Postoperative time-of-flight MRA image.

**b:** Source image corresponding to image **a**, illustrating the measurements of the caliber of STA (dotted arrow), MMA (arrowhead), and DTA (arrow).

DTA, deep temporal artery; MMA, middle meningeal artery; MRA magnetic resonance angiography; STA superficial temporal artery

**Table S1** List of previously reported moyamoya angiopathy (MMA)-related genes highlighted in this study

| **Genetic disorder** | **Gene** | **Pathology** |
| --- | --- | --- |
| ***Syndromic MMA-related genes*** | | |
| NF1 | *NF1* | RAS pathway |
| Noonan syn. | *BRAF, KRAS, PTPN11, RAF1, SOS1* | RAS pathway |
| Noonan-like syn. | *SHOC2, CBL* | RAS pathway |
| Castello syn. | *HRAS* | RAS pathway |
| Alagille syn. | *JAG1, NOTCH2* | NOTCH signal pathway |
| Robinow syn. | *ROR2* | Wnt signal pathway |
| Schimke immuno-osseous dysplasia | *SMARCAL1* | Chromatin remodeling |
| MOPD2 | *PCNT* | DNA repair |
| Seckel syn. | *ATR, RBBP8, CENPJ, CEP63, NIN* | Cell cycle |
| Grange syn. | *YY1AP1* | Chromatin remodeling |
| Sneddon syn. | *CECR1* | Inflammation |
| Aicardi–Goutières syn. | *SAMHD1* | Inflammation |
| Smooth muscle dysfunction syn. | *ACTA2* | Vascular smooth muscle cell proliferation |
| Sickle cell disease | *HBB* | Thrombosis |
| Protein S/C deficiency | *PROS1 /PROC* | Thrombosis |
| Thrombotic thrombocytopenic purpura | *ADAMTS13* | Thrombosis |
| ***Primary MMA-related genes*** | | |
| (Accompanied by achalasia) | *GUCY1A3* | NO pathway |
| (Accompanied by short stature, hypogonadism, etc.) | *BRCC3/MTCP1* | DNA repair, Angiogenesis |
|  | *DIAPH1/EVL* | Actin remodeling |
|  | *NOS3* | NO pathway |
|  | *ANO1* | Vascular smooth muscle cell phenotype |
|  | *CHD4* | Chromatin remodeling |
|  | *CNOT3* | Chromatin remodeling |
|  | *CCER2* | Unreported |

Modified from the Table in reference 32, with additional information from references 33 to 39.

**Table S2** Demographics of the enrolled participants

|  | All participants  (n=63) |
| --- | --- |
| Sex (Female) | 41 (65.1%) |
| Age at first operation, median (IQR) | 42 (33.5–50.5) |
| Hypertension | 24 (38.1%) |
| Diabetes mellitus | 3 (4.8%) |
| Dyslipidemia | 14 (22.2%) |
| Smoking | 21 (33.1%) |
| Family history of MMD | 9 (14.3%) |
| *RNF213* p.Arg4810Lys | 35 (55.1%) |
| *RNF213* Rare variants | 5 (7.9%) |
| *RNF213* Non-rare damaging variants | 0 (0.0%) |

**Table S3** Basic information on the *RNF213* rare variants that were detected

| **Position (GRCh38)** | **rsID** | **Amino Acid Change** | **AF in gnomAD** | **SIFT^1^** | **Polyphen2^2^** | **Mutation Taster^3^** | **PROVEAN^4^** | **CADD**  **score** |
| --- | --- | --- | --- | --- | --- | --- | --- | --- |
| 17:80345654 | rs761027115 | p.G2440D (c.7319G>A) | 1.4*10^-4^ | T | B | N | D | 22.5 |
| 17: 80345997 | rs138516230 | p.D2554E (c.7662C>A) | 2.9*10^-5^ | T | B | N | N | 0.118 |
| 17:80346446 | rs146486225 | p.R2704Q (c.8111G>A) | 2.4*10^-5^ | T | B | D | D | 23.3 |
| 17:80358422 | rs375097553 | p.M3666T (c.10997T>C) | 3.4*10^-4^ | D | D | D | D | 25.5 |
| 17: 80386819 | rs371441113 | p.E4950D (c.14850G>C) | 6.4*10^-5^ | T | D | N | N | 14.26 |

AF, allele frequency; gnomAD, Genome Aggregation Database; CADD, combined annotation-dependent depletion (GRCh38-v1.6); NA, not applicable; PROVEAN, Protein Variation Effect Analyzer; SIFT, Sorting Intolerant from Tolerant

^1^ D, deleterious; T, tolerated

^2^ B, benign; D, probably damaging; P, possibly damaging

^3^ A, harmless; D, probably deleterious; N, probably harmless; P, harmless

^4^ D, deleterious; N, neutral

**Table S4** Association of the *RNF213* genotype with preoperative reduction in rCBF in the MCA territory

|  |  | **GG vs GA** |  |  |  |
| --- | --- | --- | --- | --- | --- |
|  |  | **GG (n=25)** | **GA (n=33)** | ***P*-value** |  |
| rCBF* | ≥10% Reduction (n=45) | 22 (88.0%) | 23 (69.7%) | 0.098 |  |
|  | ≥20% Reduction (n=14) | 5 (20.0%) | 9 (27.3%) | 0.522 |  |
|  |  | **GG vs GG/RV vs GA** |  |  |  |
|  |  | **GG (n=22)** | **GG/RV (n=3)** | **GA (n=33)** | ***P*-value** |
| rCBF* | ≥10% Reduction (n=45) | 19 (86.4%) | 3 (100.0%) | 23 (69.7%) | 0.221 |
|  | ≥20% Reduction (n=14) | 3 (13.6%) | 2 (66.7%) | 9 (27.3%) | 0.107 |

*Compared to ipsilateral cerebellar CBF

GA, heterozygote of p.Arg4810Lys; GG, wild type of p.Arg4810Lys; rCBF, regional cerebral blood flow; RV, rare variant

**Table S5** Association of preoperative reduction in rCBF in the MCA territory and the caliber-change ratio (CCR) of STA, MMA, and DTA

|  |  | **CCR of STA,** |  | **CCR of MMA,** |  | **CCR of DTA,** |  |
| --- | --- | --- | --- | --- | --- | --- | --- |
|  |  | **median (IQR)** | ***P-*value** | **median (IQR)** | ***P*-value** | **median (IQR)** | ***P*-value** |
| rCBF* ≥10% Reduction |  |  | 0.695 |  | 0.816 |  | 0.069 |
|  | Yes (n=45) | 1.06 (0.88–1.36) |  | 1.23 (1.03–1.42) |  | 2.18 (1.45–2.73) |  |
|  | No (n=13) | 1.10 (1.00–1.28) |  | 1.23 (1.02–1.37) |  | 3.17 (2.42–3.29) |  |
| rCBF* ≥20% Reduction |  |  | 0.573 |  | 0.771 |  | 0.125 |
|  | Yes (n=14) | 1.14 (0.93–1.38) |  | 1.17 (1.01–1.34) |  | 1.77 (1.44–2.24) |  |
|  | No (n=44) | 1.06 (0.88–1.35) |  | 1.25 (1.03–1.44) |  | 2.43 (1.83–3.19) |  |

*Compared to ipsilateral cerebellar CBF

rCBF, regional cerebral blood flow

**Table S6** Basic information on rare or damaging variants detected in the 11 genes analyzed for association with postoperative collateral development

| **Gene** | **Position (GRCh38)** | **rsID** | **Amino Acid Change** | **AF in gnomAD** | **SIFT^1^** | **Polyphen2^2^** | **Mutation Taster^3^** | **PROVEAN^4^** | **CADD**  **score** |
| --- | --- | --- | --- | --- | --- | --- | --- | --- | --- |
| *ADAMTS13* | 9:133428713 | NA | p.P256S (c.766C>T) | NA | T | B | N | N | 0.421 |
|  | 9:133436943 | rs11575933 | p.P475S (c.1423C>T) | 5.8*10^-3^ | T | B | A | N | 9.307 |
|  | 9:133440409 | rs28647808 | p.P618A (c.1852C>G) | 6.1*10^-2^ | D | D | P | D | 23.7 |
|  | 9:133442619 | NA | p.R704C (c.2110C>T) | 4.1*10^-6^ | D | D | D | D | 25.3 |
|  | 9:133442676 | rs138014548 | p.Q723K (c.2167C>A) | 9.8*10^-5^ | T | B | N | N | 0.232 |
|  | 9:133443449 | rs374606481 | p.V770M (c.2308G>A) | 1.0*10^-4^ | T | P | N | N | 15.54 |
|  | 9:133445796 | rs78977446 | p.S903L (c.2708C>T) | 3.4*10^-3^ | T | B | N | N | 10.88 |
|  | 9:133455576 | rs192619276 | p.G1181R (c.3541G>A) | 1.8*10^-3^ | D | P | N | N | 7.203 |
|  | 9:133457947 | NA | p.I1254M (c.3762C>G) | NA | D | B | N | N | 3.986 |
| *ATR* | 3:142453222 | rs200490116 | p.T2556S (c.7667C>G) | 3.0*10^-4^ | T | P | D | N | 24.1 |
|  | 3:142560380 | rs781126519 | p.I475S (c.1424T>G) | 4.1*10^-6^ | T | B | N | N | 9.852 |
|  | 3:142498769 | rs193124641 | p.K1796Q (c.5386A>C) | 1.2*10^-5^ | T | B | D | N | 22.4 |
|  | 3:142513527 | rs138350940 | p.G1539S (c.4615G>A) | 7.3*10^-5^ | T | B | D | N | 22.5 |
|  | 3:142563077 | rs146405935 | p.R109W (c.325C>T) | 1.3*10^-3^ | D | D | D | N | 24.7 |
| *CBL* | 11: 119285537 | rs772002109 | p.G638R (c.1912G>A) | 1.2*10^-5^ | D | B | D | N | 24.2 |
|  | 11:119298451 | rs2229073 | p.P782L (c.2345C>T) | 8.0*10^-4^ | D | P | D | N | 23.9 |
|  | 11: 119285268 | rs17848897 | p.P548L (c.1643C>T) | 6.5*10^-5^ | D | D | D | D | 26.4 |
| *CENPJ* | 13: 24905576 | rs144938364 | p.T821M (c.2462C>T) | 1.4*10^-3^ | T | B | N | N | 0.672 |
|  | 13: 24906818 | NA | p.S407Y (c.1220C>A) | NA | D | P | N | N | 21.9 |
|  | 13: 24912958 | rs116981543 | p.N23S (c.68A>G) | 6.7*10^-3^ | T | B | N | N | 8.486 |
|  | 13: 24909892 | rs150932292 | p.T255A (c.763A>G) | 1.6*10^-3^ | T | B | N | N | 9.484 |
|  | 13: 24905888 | NA | p.S717L (c.2150C>T) | NA | T | B | N | N | 4.454 |
|  | 13: 24911971 | rs781695862 | p.M181I (c.543G>T) | 1.2*10^-5^ | D | B | N | N | 18.75 |
|  | 13: 24905696 | rs201828176 | p.S781L (c.2342C>T) | 6.9*10^-5^ | T | B | N | N | 7.422 |
|  | 13: 24906855 | NA | p.G395S (c.1183G>A) | NA | T | B | N | N | 15.82 |
|  | 13: 24905440 | NA | p.M866I (c.2598G>A) | NA | D | B | D | N | 22.1 |
| *NF1* | 17: 31340631 | NA | p.I2350V (c.7048A>G) | NA | T | B | D | N | 15.7 |
|  | 17: 31225182 | rs146051850 | p.M645V (c.1933A>G) | 1.2*10^-3^ | T | B | N | N | 16.57 |
|  | 17: 31358607 | NA | p.T2700P (c.8098A>C) | NA | T | B | D | N | 22.9 |
|  | 17: 31326144 | rs773378630 | p.E1720D (c.5160G>T) | 4.1*10^-6^ | T | B | D | N | 12.64 |
|  | 17: 31260383 | rs746994734 | p.I1482T (c.4445T>C) | 2.4*10^-5^ | D | B | D | D | 24.4 |
| *NIN* | 14: 50770509 | rs188064359 | p.R438Q (c.1313G>A) | 1.0*10^-4^ | T | B | N | N | 2.059 |
|  | 14: 50752531 | rs149669464 | p.R1646H (c.4937G>A) | 1.8*10^-3^ | T | B | N | N | 1.891 |
| *NOS3* | 7: 151010136 | rs759020736 | p.V845G (c.2534T>G) | 1.0*10^-4^ | T | B | D | N | 22.6 |
|  | 7: 150996479 | rs200136447 | p.G116S(c.346G>A) | 3.0*10^-4^ | T | B | N | N | 4.591 |
|  | 7: 151010723 | rs760761084 | p.R938W (c.2812C>T) | NA | D | D | D | D | 32 |
| *NONTCH2* | 1: 119955217 | rs74882029 | p.I681N (c.2042T>A) | 3.0*10^-4^ | T | B | N | N | 24 |
|  | 1: 119929089 | rs75423398 | p.R1260H (c.3779G>A) | 1.4*10^-3^ | T | B | D | D | 22.4 |
|  | 1: 119937372 | NA | p.S1144R (c.3432C>A) | NA | D | D | D | D | 25.5 |
|  | 1: 119919409 | rs201996575 | p.R1895H (c.5684G>A) | 8.5*10^-5^ | D | B | D | D | 24.2 |
|  | 1: 119922384 | rs60854092 | p.I1689F (c.5065A>T) | 2.0*10^-3^ | T | B | D | N | 19.01 |
|  | 1: 119916499 | rs150516342 | p.V2075M (c.6223G>A) | 1.3*10^-3^ | T | B | N | N | 16.86 |
|  | 1: 119925513 | rs777091426 | p.R1435W (c.4303C>T) | 1.6*10^-5^ | D | D | D | N | 25.4 |
|  | 1: 119941691 | rs201100122 | p.P939L (c.2816C>T) | 1.0*10^-4^ | D | B | D | D | 18.82 |
| *PCNT* | 21: 46381695 | rs143253402 | p.G1056D (c.3167G>A) | 7.7*10^-5^ | T | B | N | N | 14.1 |
|  | 21: 46436082 | rs146657011 | p.A2977V (c.8930C>T) | 3.0*10^-4^ | D | B | N | N | 10.37 |
|  | 21: 46412874 | rs145119952 | p.A2011V (c.6032C>T) | 2.0*10^-4^ | D | B | N | N | 8.982 |
|  | 21: 46326566 | rs143870030 | p.A82T (c.244G>A) | 2.0*10^-4^ | T | B | N | N | 2.444 |
|  | 21: 46411720 | rs746468689 | p.R1883W (c.5647C>T) | 2.5*10^-5^ | T | B | N | N | 6.498 |
|  | 21: 46411783 | rs200426591 | p.A1904T (c.5710G>A) | 2.3*10^-3^ | T | B | N | N | 1.751 |
|  | 21: 46418216 | rs537029143 | p.E2312K (c.6934G>A) | 8.1*10^-6^ | D | D | D | D | 25.3 |
|  | 21: 46389289 | rs199787861 | p.E1233A (c.3698A>C) | 2.0*10^-4^ | D | P | N | D | 24.1 |
|  | 21: 46381748 | rs200174202 | p.R1074W (c.3220C>T) | 2.0*10^-4^ | D | B | N | N | 6.34 |
|  | 21: 46416177 | NA | p.Q2087E (c.6259C>G) | 4.1*10^-6^ | T | B | N | N | 12.79 |
|  | 21: 46441017 | rs765572416 | p.R3186W (c.9556C>T) | 7.7*10^-5^ | D | D | N | D | 23.3 |
|  | 21: 46411414 | rs200137805 | p.G1781R (c.5341G>A) | 1.0*10^-4^ | T | B | N | N | 7.193 |
|  | 21: 46389307 | rs201315836 | p.R1239H (c.3716G>A) | 2.0*10^-4^ | T | B | N | N | 25.1 |
|  | 21: 46411616 | rs201943167 | p.E1848G (c.5543A>G) | 2.1*10^-5^ | D | P | N | D | 23.7 |
| *ROR2* | 9: 91733124 | rs188376581 | p.R312H (c.935G>A) | 3.0*10^-4^ | D | B | D | N | 20.5 |
|  | 9: 91724738 | rs142386294 | p.A586T (c.1756G>A) | 3.0*10^-4^ | T | B | D | N | 13.87 |
|  | 9: 91724282 | rs56231927 | p.R738C (c.2122C>T) | 2.0*10^-4^ | D | D | D | D | 30 |
|  | 9: 91724564 | rs55798732 | p.D644N (c.1930G>A) | 2.0*10^-4^ | D | D | D | D | 27.1 |
|  | 9: 91775770 | rs201991252 | p.G49V (c.146G>T) | 9.7*10^-5^ | D | B | N | N | 17.71 |
|  | 9: 91726620 | rs149842671 | p.A436V (c.1307C>T) | NA | T | B | N | N | 13.33 |
| *YY1AP1* | 1: 155660882 | rs150614243 | p.R332Q (c.995G>A) | 4.0*10^-4^ | T | B | D | N | 1.91 |
|  | 1: 155660774 | NA | p.R368P (c.1103G>C) | NA | T | B | N | D | 0.24 |
|  | 1: 155688244 | rs758007166 | p.L74X (c.221T>A) | 6.4*10^-5^ | NA | NA | N | NA | 33 |

AF, allele frequency; gnomAD, Genome Aggregation Database; CADD, combined annotation-dependent depletion (GRCh38-v1.6); NA, not applicable; PROVEAN, Protein Variation Effect Analyzer; SIFT, Sorting Intolerant from Tolerant

^1^ D, deleterious; T, tolerated

^2^ B, benign; D, probably damaging; P, possibly damaging

^3^ A, harmless; D, probably deleterious; N, probably harmless; P, harmless

^4^ D, deleterious; N, neutral

**References**

32. Koizumi A, Kobayashi H, Hitomi T, Harada KH, Habu T, Youssefian S. A new horizon of moyamoya disease and associated health risks explored through RNF213. *Environmental Health and Preventive Medicine*. 2016;21:55-70. doi: 10.1007/s12199-015-0498-7

33. Kossorotoff M, Tournier-Lasserve E, Herve D, Guey S. Moyamoya disease and syndromes: from genetics to clinical management. *The Application of Clinical Genetics*. 2015:49. doi: 10.2147/tacg.s42772

34. Kundishora AJ, Peters ST, Pinard A, Duran D, Panchagnula S, Barak T, Miyagishima DF, Dong W, Smith H, Ocken J, et al. <i>DIAPH1</i> Variants in Non–East Asian Patients With Sporadic Moyamoya Disease. *JAMA Neurology*. 2021;78:993. doi: 10.1001/jamaneurol.2021.1681

35. Guey S, Hervé D, Kossorotoff M, Ha G, Aloui C, Bergametti F, Arnould M, Guenou H, Hadjadj J, Dubois Teklali F, et al. Biallelic variants in NOS3 and GUCY1A3, the two major genes of the nitric oxide pathway, cause moyamoya cerebral angiopathy. *Human Genomics*. 2023;17. doi: 10.1186/s40246-023-00471-x

36. Pinard A, Ye W, Fraser SM, Rosenfeld JA, Pichurin P, Hickey SE, Guo D, Cecchi AC, Boerio ML, Guey S, et al. Rare variants in ANO1, encoding a calcium-activated chloride channel, predispose to moyamoya disease. *Brain*. 2023. doi: 10.1093/brain/awad172

37. Guo D-C, Duan X-Y, Regalado ES, Mellor-Crummey L, Kwartler CS, Kim D, Lieberman K, De Vries BBA, Pfundt R, Schinzel A, et al. Loss-of-Function Mutations in YY1AP1 Lead to Grange Syndrome and a Fibromuscular Dysplasia-Like Vascular Disease. *The American Journal of Human Genetics*. 2017;100:21-30. doi: 10.1016/j.ajhg.2016.11.008

38. Mukawa M, Nariai T, Onda H, Yoneyama T, Aihara Y, Hirota K, Kudo T, Sumita K, Maehara T, Kawamata T, et al. Exome Sequencing Identified CCER2 as a Novel Candidate Gene for Moyamoya Disease. *J Stroke Cerebrovasc Dis*. 2017;26:150-161. doi: 10.1016/j.jstrokecerebrovasdis.2016.09.003

39. Pinard A, Guey S, Guo D, Cecchi AC, Kharas N, Wallace S, Regalado ES, Hostetler EM, Sharrief AZ, Bergametti F, et al. The pleiotropy associated with de novo variants in CHD4, CNOT3, and SETD5 extends to moyamoya angiopathy. *Genetics in Medicine*. 2020;22:427-431. doi: 10.1038/s41436-019-0639-2
